# An mHealth App to Support Caregivers in the Medical Management of Their Child With Cancer: Beta Stage Usability Study

**DOI:** 10.2196/52128

**Published:** 2024-10-17

**Authors:** Emily L Mueller, Anneli R Cochrane, Madison E Campbell, Sarah Nikkhah, Richard J Holden, Andrew D Miller

**Affiliations:** 1 Pediatric Accelerator for Careers Engaged in Research Indiana University School of Medicine Indianapolis, IN United States; 2 Section of Pediatric Hematology Oncology Department of Pediatrics Indiana University School of Medicine Indianapolis, IN United States; 3 Indiana University-Purdue University Indianapolis Indianapolis, IN United States; 4 Human-Centered Computing Department School of Informatics and Computing Indiana University-Purdue University Indianapolis Indianapolis, IN United States; 5 Health & Wellness Design School of Public Health Indiana University-Bloomington Bloomington, IN United States

**Keywords:** oncology, supportive care, mHealth, children, caregivers, mobile phones

## Abstract

**Background:**

Previous research demonstrated that caregivers of children with cancer desired a mobile health (mHealth) tool to aid them in the medical management of their child. Prototyping and alpha testing of the Cope 360 app (Commissioning Agents, Inc) resulted in improvements in the ability to track symptoms, manage medications, and prepare for urgent medical needs.

**Objective:**

This study aims to engage caregivers of children with cancer in beta testing of a smartphone app for the medical management of children with cancer, assess acceptance, identify caregivers’ perceptions and areas for improvement, and validate the app’s design concepts and use cases.

**Methods:**

In this pilot, study caregivers of children with cancer used the Cope 360 mHealth app for 1 week, with the goal of daily logging. Demographics and a technology acceptance survey were obtained from each participant. Recorded semistructured interviews were transcribed and analyzed iteratively using NVivo (version 12, QSR International) and analyzed for information on usage, perceptions, and suggestions for improvement.

**Results:**

A total of 10 caregivers participated in beta testing, primarily women (n=8, 80%), married, with some college education, and non-Hispanic White (n=10, 100%). The majority of participants (n=7, 70%) had children with acute lymphocytic leukemia who were being treated with chemotherapy only (n=8, 80%). Overall, participants had a favorable opinion of Cope 360. Almost all participants (n=9, 90%) believed that using the app would improve their ability to manage their child’s medical needs at home. All participants reported that Cope 360 was easy to use, and most would use the app if given the opportunity (n=8, 80%). These values indicate that the app had a high perceived ease of use with well-perceived usefulness and behavioral intention to use. Key topics for improvement were identified including items that were within the scope of change and others that were added to a future wish list. Changes that were made based on caregiver feedback included tracking or editing all oral and subcutaneous medications and the ability to change the time of a symptom tracked or medication administered if unable to do so immediately. Wish list items included adding a notes section, monitoring skin changes, weight and nutrition tracking, and mental health tracking.

**Conclusions:**

The Cope 360 app was well received by caregivers of children with cancer. Our validation testing suggests that the Cope 360 app is ready for testing in a randomized controlled trial to assess outcome improvements.

## Introduction

The necessary changes a parent or guardian has to make in caregiving when a child is diagnosed with cancer are immense and overwhelming [1-3]. In the home setting, caregivers must oversee complicated symptom management and medication administration needs. One viable avenue to address the complex needs of caregivers of children with cancer is through mobile health (mHealth) technology, defined as the application of mobile or wireless communication technologies to health and health care [4]. Many apps have been developed to address the needs of children and adolescents with cancer, yet few focus on the unique needs of caregivers who are overseeing the medical management of a child with cancer [5]. mHealth tools have the unique ability to support caregivers through their portability and ability to share data between multiple parties in real time.

Caregivers of children with cancer are known to use mHealth tools and in a recent survey study, the majority desired a tool to help with medical management [6]. Specifically, they desired a tool that would help with medical knowledge, symptom tracking, and medication reminders [6]. To ensure an effective tool is developed to respond to the gaps identified by caregivers, it is imperative to study and incorporate intended end users’ specific perspectives and needs during mHealth tool development [7-9]. Involving end users increases the likelihood the app will both work for them and be used by them.

Developers and researchers of mHealth technology must also address the future acceptance of their product through direct interaction with the end users during the development process. Nadal et al [10] explored the important differences in acceptance versus acceptability and proposed the Technology Acceptance Lifecycle model, which highlights the evolving nature of technology acceptance across different stages of the user journey with the technology tool [10]. The Technology Acceptance Lifecycle explores the preuse acceptability, initial use acceptance, and sustained use acceptance which align with a shift in initial use acceptance from preadoption to postadoption of the tool in use.

To understand and address the needs of caregivers, our team engaged directly with the intended users to create the Cope 360 mHealth tool (Commissioning Agents, Inc). Thus far, caregivers have been involved in the co-design, prototyping, and initial refinement [11,12]. The objective of this study is to evaluate the initial use acceptance, and functionality of the Cope 360 app in a week-long trial by caregivers of children with cancer. The significance of this work is to demonstrate the importance of including the intended end users in acceptance testing outside the research environment in order to inform further refinements of mHealth tools such as Cope 360.

## Methods

### Study Design

In this pilot study, we performed qualitative interviews and used a validated acceptance survey to engage directly with end users (ie, caregivers of children with cancer) to test an app to support caregivers in the medical management of their child with cancer. There were 3 phases of this project: prototyping of the app (phase 1), followed by alpha testing directly with caregivers (phase 2), and finally, initial use beta testing with caregivers (phase 3), which we evaluate here. Alpha and beta testing are validation methodologies that help researchers and designers assess the initial use acceptance and perceptions of end users. These tests provide opportunities for refinement before launching the product on a larger scale and result in greater success of the product for regular use [13]. In this final phase (phase 3), we collected measures of acceptance of the technology tool and performed qualitative semistructured interviews between May 2021 and October 2021.

### Brief Summary of Cope 360 Features

The overall intent of the app is to assist caregivers in the medical management aspects of their child with cancer while they are outside of the hospital setting. It was not intended to be used while patients were being actively treated by a medical professional or under the direct care of an oncologist (such as during hospital admissions for therapy). After developing and prototyping the app, known as Cope 360, we performed alpha testing of the app with 6 nurse coordinators and 8 caregiver participants [12]. Alpha testing of Cope 360 resulted in improvements in clarity of medical information and terminology, improvement in the design of tasks, and tracking of symptoms including adjusting the look and feel of certain buttons and changing the visual graph used to monitor symptoms to include date anchors.

**Figure 1:**
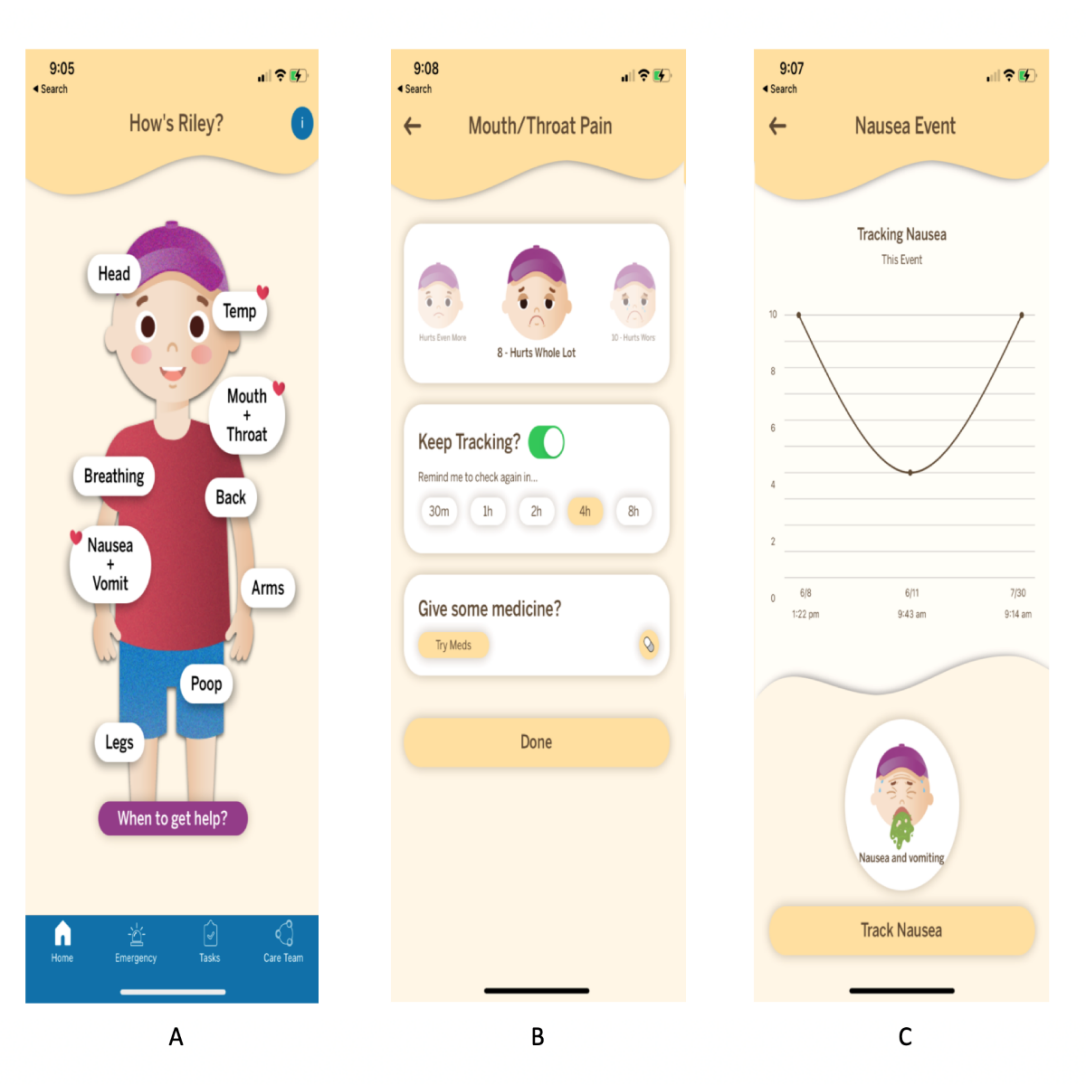
Screenshots of Cope 360 key screens: (A) home screen, (B) symptom tracking, and (C) symptom monitoring.

The symptom tracking feature is located on the home screen, where there is a cartoon representation of the patient that can be personalized by sex and 3 skin colors. The three key functions of the app are (1) symptom tracking, (2) medication management, and (3) emergency preparedness. The symptom tracking had nine options for tracking, including (1) temperature, (2) breathing, (3) nausea and vomiting, (4) poop, and pain in the following areas: (5) head, (6) mouth and throat, (7) back, (8) arms, and (9) legs. Each symptom has an individualized tracking scale based on previously published or validated scales. The temperature tracking provides directed feedback based on the temperature input from the caregiver. The medication management portion includes all current medications the patient is taking including oral chemotherapy and supportive medications that are entered by the nurse coordinators. The emergency preparedness plan allows the caregiver to create, practice, and enact a plan for seeking care for an urgent medical issue. Screenshots of the app key screens are included in Figure 1. The Cope 360 app was a fully functioning app that was downloaded by the caregiver onto either Apple or Android smartphones using a web-based download link provided to the participants upon consent.

### Study Population

Participants were caregivers of a child with cancer (the child had to be younger than 21 years), had adequate English-language proficiency with grossly normal cognitive function, and had a child who was currently receiving cancer therapy at Riley Hospital for Children in Indianapolis, Indiana, and at least 1 month had passed after the initial diagnosis. We included caregivers of children up to the age of 21 due to most of our Children’s Oncology Group clinical trials allowing for patients up to that age. Caregivers were contacted by phone for recruitment and interview scheduling; interviews were conducted and recorded over Zoom (Zoom Video Communications) videoconferencing due to COVID-19 precautions.

### Measurements

For the beta test, demographic information was collected from participants using a web-based survey, including caregiver sex, age, race and ethnicity, marital status, yearly household income, and education. Additional questions included the relationship of the caregiver to the child, the child’s type of cancer, and the child’s therapy. After enrollment, the physician on the study team (ELM) reviewed the electronic medical record of the patient and added the documented medications into the Cope 360 app; caregivers were able to confirm and correct all medications. The caregivers were then asked to use the app for either actual or simulated situations for a period of 1 week, logging in at least once per day. At the end of the week, they were asked to participate in an audio-recorded semistructured qualitative interview by the research assistant (ARC) during which caregivers were asked open-ended questions including their use of the app, any problems they experienced, recommendations for improvement, and how useful they found the app during the week. See Multimedia Appendix 1 for semistructured interview guide.

At the end, the participants completed a web-based survey that included the modified Technology Acceptance Model (TAM) to measure their perceptions and acceptance of the app [14-16]. TAM is a behavioral model of end-user acceptance of new technologies. The use of TAM in the health care field has been relatively widespread and justified [16]. In this model, 3 factors are needed to explain and predict the actual use of information technology:

- Perceived usefulness: the end user’s perception of whether the tool will accomplish its intended purpose.
- Perceived ease of use: the end user’s perception of how easy it is to navigate within a tool and their reactions to the overall “look or feel” of the interface.
- Behavioral intention to use: the end user’s perceived likelihood that they will engage and use a given tool.

Due to the small number of participants, the Likert scale categories collapsed into agree, neutral, and disagree. Items for this questionnaire were adapted from a study conducted by Venkatesh and Davis [17], which showed high reliability and strong construct validity.

### Ethical Considerations

Development and refinement of the app were made possible through a partnership with Coactive Business Solutions of Indianapolis, Indiana. The Indiana University institutional review board approved this study (1903250567). Potential participants received a study information sheet via email that described the project and their ability to withdraw at any point in the interview. This was reviewed and then they consented to enrollment verbally. All data collected from caregivers were saved on a secure, HIPAA (Health Insurance Portability and Accountability Act) safe server with access only by the research team. Caregiver participants were compensated with a US $60 gift card for the 1-week use of the Cope 360 app, survey, and semistructured interview.

### Analysis

Characteristics of study participants were summarized by frequency and range. Descriptive statistics of the acceptance survey were performed. To analyze the semistructured interview data, the research team focused on both (1) the usability and functioning of the app and (2) evaluated key caregiver-derived topics related to future improvement. The evaluation of the usability and function included open-ended questions about the following: app usage by the caregiver, including log-in and account creation, symptom tracking experience, perceptions of emergency planning, overall experience, and suggestions for future improvements.

The team conducted deductive and inductive analysis on the interview transcripts. Caregiver semistructured interviews were transcribed by a HIPAA-compliant service and then analyzed using NVivo (version 12; QSR International). First, an initial codebook was created deductively using the interview question topics listed above as the primary themes. In this study, the main themes were focused on the user experience during the beta testing phase of the Cope 360 app, with an emphasis on positive attributes of usability and function and key elements for improvement. Transcription and coding were performed as interviews were conducted and interviews continued until no new information was gathered and thematic saturation was achieved [18,19]. Two team members (ARC and MC) independently reviewed each transcript and assigned codes based on themes using an initial codebook. The codebook was revised based on new themes that emerged through data review [18,19]. The main inductive codes that were added during iterative analysis were the identification of issues with the intended app features, such as the lack of push notifications. A final review was performed by 3 team members (ARC, MC, and ELM) until an agreement on codes and themes was attained. A total of 58 codes were in the finalized codebook.

## Results

### Demographic Information of Study Participants

A total of 23 caregivers were contacted with 10 caregivers (females: n=8, 80% and males: n=2, 20%) participating in beta testing. All were married parents, and non-Hispanic White (n=10, 100%). Seven (70%) had children with acute lymphocytic leukemia and 3 (30%) had solid tumors. The majority had children being treated with chemotherapy only (n=8, 80%), 1 patient being treated using both chemotherapy and radiation and 1 (10%) with another form of treatment. All caregivers had at least some college education. All caregivers reported a yearly household income of at least US $50,000 to US $74,999 (Table 1).

**Table 1:** Demographic characteristics of the Cope 360 app beta testing participants (N=10).

Characteristics				Caregivers
**Caregiver sex, n (%)**				
	Male		2 (20)	
	Female		8 (80)	
Caregiver age (years; n=8), median (IQR) 37 (34-43)				38 (8.25; 33-47)
Child age (years; n=10), median (IQR) 6 (4-7)				6 (2.75; 2-9)
**Caregiver race and ethnicity, n (%)**				
	Non-Hispanic White		10 (100)	
**Type of cancer, n (%)**				
	Acute lymphoblastic leukemia		7 (70)	
	Solid tumor		3 (30)	
**Type of therapy, n (%)**				
	Chemotherapy only		8 (80)	
	Chemotherapy and radiation		1 (10)	
	Other		1 (10)	
**Type of caregiver, n (%)**				
	Parent		10 (100)	
**Caregiver marital status, n (%)**				
	Married		10 (100)	
**Caregiver yearly household income (US $), n (%)**				
	Less than 49,999	0 (0)		
	50,000-74,999	1 (10)		
	75,000-99,999	4 (40)		
	100,000-150,000	1 (10)		
	Greater than 150,000	3 (30)		
	Prefer not to answer	1 (10)		
**Caregiver education, n (%)**				
	Less than high school	0 (0)		
	High school or GED^a^	0 (0)		
	Some college	1 (10)		
	College graduate	6 (60)		
	Graduate degree	3 (30)		

^a^GED: general educational development.

### Participant Initial Use Acceptance

**Figure 2:**
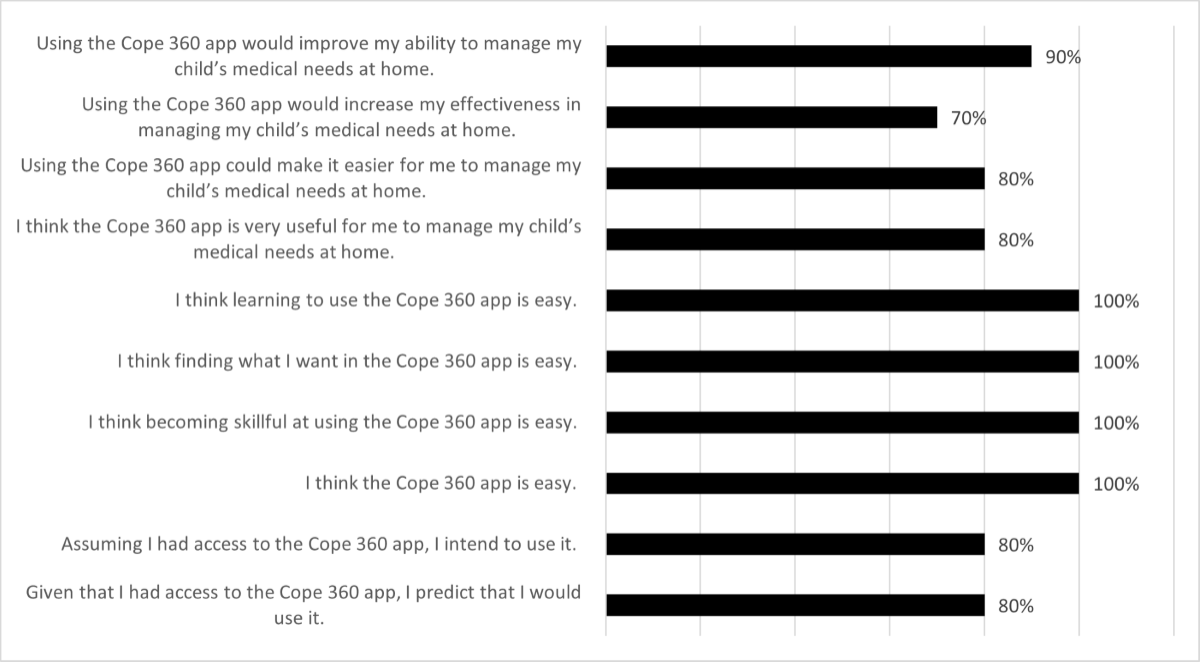
Technology Acceptance Model agreement ratings by participants.

A summary of participants’ TAM overall favorability rating is presented in Figure 2. Overall, participants had a favorable opinion of Cope 360. Almost all (n=9, 90%) felt that using the app would improve their ability to manage their child’s medical needs at home. The majority agreed that using the app would increase their effectiveness (n=7, 70%) and make it easier for them to manage their child’s needs at home (n=8, 80%). All participants felt that Cope 360 was easy to use. Most felt they would use the app if given the opportunity (n=8, 80%) with neutral (n=1, 10%) and disagree (n=1, 10%). These scores indicate that the app had a high perceived ease of use with good perceived usefulness and behavioral intention to use.

### Participant Interviews

Analysis of the participant interviews revealed several general themes related to the user experience including initial setup, overall experience, experience with logging in, creating an account, symptom tracking, emergency planning, and a concluding category with questions such as future use of the app if publicly available and caregiving apps currently used.

### Perceptions of Initial Set-Up

Participants were asked to use the app at least once a day throughout their week of using the app. When questioned about their actual usage, 9 (90%) participants said they used it every day or tried to use it every day; 1 (10%) participant said they used it about 10 times during the 7-day period. When asked about their experience logging in, 9 (90%) stated that they had no difficulties logging into the app. Two (20%) of the participants mentioned they had difficulties figuring out how to log out. One participant suggested moving the logout button to a more obvious area. All participants mentioned that creating a caregiver account as well as an account for their child was easy. When asked if they added other caregivers to their caregiver team, 3 (30%) did perform this task with no difficulty, while 7 (70%) did not add anyone.

### Perception of Symptom Tracking

All participants used the symptom tracking feature of the app when asked what symptoms they had tracked: 8 (80%) tracked poop, 7 (70%) tracked nausea or vomiting, 4 (40%) tracked head pain, 3 (30%) tracked temperature, 2 (20%) tracked arm pain, and 3 (30%) tracked leg pain. When questioned on ease of use, all participants thought symptom tracking was easy to use, with 1 (10%) participant suggesting that they would like the ability to edit previous entries and another mentioning that they had some confusion about the meaning of the nausea or vomiting scale. Features of symptom tracking that were viewed positively included reminders (n=4, 40%), scales (n=4, 40%), and graphing (n=2, 20%). Noted issues or suggestions while using symptom tracking included not receiving notifications (n=9, 90%) and wanting to see more symptoms added (n=1, 10%).

Pulsing heart on symptom tracking shows that a symptom is actively being tracked. When asked if participants preferred a different method for showing they are tracking a symptom, 8 (80%) participants said they had no preference for a different method, while 1 (10%) suggested making it a color scheme instead of a pulsing icon, and 1 (10%) suggested making the icon a clock or timer since there is a time component to the symptom tracking. Finally, when asked about how they stopped tracking a symptom, 4 (40%) participants stated they did not stop tracking, 3 (30%) said stopping symptom tracking was easy for them and made sense, and 3 (30%) suggested other ways to stop tracking, such as an alarm clock you can snooze (n=1, 10%), and moving the tracking on or off to the overview page instead of having to enter a final symptom (n=2, 20%).

### Perception of Emergency Planning

Participants were then asked about their experience with the emergency planning part of the app. Eight participants (80%) stated that they set up their emergency plans, but 2 (20%) did not set up the emergency plan. Of those who set it up, all thought it was easy to set up and helpful to them. A total of 6 participants (60%) mentioned that they experienced no issues with their emergency plan. One participant (10%) expressed concern about the phone number to the hospital in their emergency plan and stated it would be helpful to know if it goes to someone directly when it is off hours or if it is voicemail regulated, and 1 participant (10%) was unsure of how to execute the plan after setting it up. Included in the app is an electronic “card” that a caregiver can reference when in contact with a health care provider not familiar with caring for a child with cancer, and 9 participants (90%) felt there was nothing more necessary to add to that section.

### Overall Perception of the Cope 360 App

All participants said that they would use or would possibly use Cope 360 if it was publicly available. Those who would possibly use it said that it would depend on whether their child was experiencing symptoms or not. Eight (80%) participants stated that the app assisted with or potentially assisted with the care of their child.

### Key Topics for Improvement and Summary of Caregiver Feedback

In Table 2, we present the key topics for improvement along with key quotes from caregivers. Regarding medications, caregivers desired the ability to track and edit medication names or doses and the ability to change the time of a tracked symptom or medication if they did not perform the tracking in real-time. Additionally, they requested to be able to have a notes section to keep track of thoughts and ideas related to the child’s clinical experience. They also desired tracking capabilities for skin changes, weight or nutrition, and mental health.

**Table 2:** Key quotes from Cope 360 improvement suggestions.

Themes	Examples of codes
Tracking all medications	“For medications when we give him, there's not really a journal or a diary that we have the option on this app to put him in. It's more like you have a headache, okay, you should take Tylenol, or you can take this. It gives you the options. Whereas I give him daily medicine and I need to be able to be like, this is the medicine I'm giving him at this time. You know what I mean?” [Mother of a 6-year-old boy with neuroblastoma] “I was thinking that like if maybe his daily meds that he has to take, maybe that don’t like have to do with any of these other things like nausea and vomiting and that sort of thing. If there was a way to track that he had taken those, that would be helpful. Like administering his daily medications, knowing that we took those somehow that would be helpful in there. For me at least. I don’t know about everybody else. And then I was thinking that somewhere on there, if there was an area to track, maybe some other symptoms maybe just put like other on there.” [Mother of a 5-year-old boy with ALL^a^]
Editing medications	“In the patient info, I know that [a healthcare provider] had to enter in the medications. I do think that's something that would be also be helpful if that was like either up-to-date or that I could edit them or something because I think that comes up a lot. Every time we come to clinic, we are talking about medications yesterday. We had a conversation about medications. And so I think if that was up-to-date or easier to edit would be helpful to just like [update] that record. Or if they were constantly, I don't know, it seems like a lot of work for them to constantly update everybody - but yeah, if I could add in stuff here that would be helpful.” [Father of a 2-year-old girl with ALL]
Changing the time of tracked symptom or medication	“There are parts of it that I would maybe change in that like I can’t manually input the time post hoc after the event. It would make it appear as though it just happened then versus sometimes it’s maybe you’re somewhere else and they have to go to the bathroom, or you take their temperature but then you don’t write it down for an hour or so. So maybe having something where you could actually like input the time or create kind of a note within the event.” [Father of a 3-year-old girl with ALL]
Adding a notes section	“Well, I like the reminders and that you can see like where you were last time when you go back in. I thought that was good. It might have been good place to have a place to put some notes in because it’s very like, just click on a picture. I didn’t think of this until now, but it might be handy to be able to put a note in if you wanted to. [Mother of a 7-year-old girl with ALL] “So maybe having something where you could actually like input the time or create kind of a note within the event. And if that is a possibility that would, I didn't see it, but that would be maybe one thing that I would improve upon, kind of having a note section to kind of further explicate or be able to manually say hey you know, this happened at noon.” [Father of a 3-year-old girl with ALL]
Monitoring skin changes	“Obviously, I just primarily know from our experience and then the experience of some other cancer families that we've gotten to know. Rashes are something that pops up, I wouldn't say fairly regularly, but it has popped up. She's probably had 10 different rashes over the last eight months, so that may be rashes or bruising. Maybe if there was a health tracker for skin where you can then get into, is it rashes and bruising? … I would maybe add another health tracker and just maybe call it skin, and then you have it so you could put in if there was a rash, if there was a cut, if there was a bruise because the rashes, especially with all the medicines pop up quite a bit. Then the bruising, especially when blood counts are low, immunity is low, the bruising can become a pretty significant symptom.” [Mother of a 9-year-old girl with ALL]

^a^ALL: acute lymphoblastic leukemia.

## Discussion

### Principal Findings

In the initial use beta testing of an app to support caregivers in the medical management of children with cancer, our team found that the Cope 360 app was well received by caregivers and offers the potential to impact the outpatient medical care of children with cancer. Specifically, caregivers were able to successfully track the most common symptoms experienced by their child with cancer. Initial use beta testing was able to identify a limitation and several key areas for refinement based on caregivers’ usage and needs. Specifically, it was identified that having access to and the ability to adjust medications was desired by caregivers therefore this was prioritized as an improvement in the Cope 360 app refinement. The next step for the Cope 360 app will be to test the feasibility and sustained use acceptance over a longer period with additional emphasis on how a tool to support caregivers could improve their perceptions of their medically focused caregiving roles.

### Beta Testing Success and Tradeoffs

The success of the beta testing of the Cope 360 app likely was impacted by the continuous engagement of key stakeholders from conception through prototyping and refining. During the initial work with co-design and creation, we had focused on preparing caregivers for when medical emergencies arose, but the insight and contribution of caregivers helped clarify that providing tracking and overall medical management was integral [12,20]. Allowing caregivers to directly interact with the app outside of the formative testing sessions shed light on the participants' initial use acceptance and created a great opportunity for wider exposure to how the app could be used.

One key challenge in beta testing that was noted, as compared with previous prototyping and alpha testing, was the gap in clarity of the intended notifications and features of the app. For example, many caregivers did not know that they were supposed to be receiving push notifications for medications, which were found to be not working well. One recommendation to overcome this challenge would be to improve clarity for first-time app users by offering a comprehensive review of the app features [12]. Another opportunity to overcome this challenge is to create a notifications section within the user interface that would alert the user to the intention to receive notifications.

### Changes Made and Future Directions for Cope 360

Engagement with end users demonstrated the need for further refinement to address the desires of caregivers of children with cancer. The changes we were able to make to the app included the end user (eg, caregivers) are now able to adjust medication names and doses. They are also able to adjust the time of the medication administration if they do not document it in real-time. This was important to caregivers and our team because it allows caregivers to continue to use the app even during holds or adjustments of medications due to patient illness or based on chemotherapy adjustment strategies for toxicity. This ability to adjust medications adds an additional layer of protection to ensure accurate medication dosages in that both health care professionals will be capable of inputting the medications and caregivers will be able to adjust in real time. This is especially important since holds or adjustments can occur overnight or on a weekend when team members are not available. There were several desires for changes by caregivers that were not within the scope of the current project budget but will be incorporated into future research endeavors including adding a notes section and monitoring for skin changes, weight or nutrition, and mental health.

The Cope 360 app performed well in initial use acceptance and has the ability to meaningfully impact both patient and caregiver outcomes. However, before deploying widely, future research on the Cope 360 app will be needed to explore feasibility, usability, and caregiver outcomes using mixed methods to get a more robust understanding of the experience of caregivers as they manage their child’s medical needs in the community setting. Building off Van Houtven organizing framework for caregiver interventions [21], our research team plans to evaluate 3 caregiver outcomes and engage with end users through semistructured interviews. The elements of the Van Houtven framework that we believe the Cope 360 app can address are clinical knowledge and caregiver self-efficacy. Therefore, we will be assessing caregiver self-efficacy [22], mastery of caregiving [23], and caregiver stress [24]. We also intend to dive deeper into the feasibility of this app in real-life setting over a prolonged period of time by evaluating which symptoms are most commonly tracked and the frequency of app usage.

### Recommendation for mHealth App Development

The process of co-design, creation, and refinement of an mHealth tool holds many lessons for health care professionals interested in engaging in the design and use of mHealth tools for their patient populations. First, incorporating the end users from inception highlighted their unique needs and desires. These were then brought to the forefront of all testing. Evaluation in a controlled research environment allowed an increased understanding of the users’ needs related to the interface of the tool. However, evaluation of practical acceptability was best achieved through the initial use testing period. The challenge we found was the missed opportunity for feedback on features the participants were unaware of. There are several ways to overcome this challenge including through a more detailed orientation process with the mHealth tool prior to the initial use testing and through period monitoring of use during the trial period.

### Limitations

There are several limitations to the initial use testing of the Cope 360 app in this study. The primary limitation was the small sample size which limited the ability to fully evaluate the TAM. Therefore, general statements about acceptability were included. The qualitative feedback obtained through this sample was robust and covered many key features and future design suggestions. Yet our team appreciates that the sample was lacking in diversity, which may have highlighted other findings not included in this analysis. This study occurred at a single institution with the investment of the study team to incorporate the patient’s current medications into the app upon enrollment. Currently, the app is designed to be used by caregivers but has medications generated by health care providers.

### Conclusions

The initial use evaluation of the Cope 360 app by caregivers of children with cancer confirmed its acceptability and usability of aid in medical management in the home setting. The next phase will be to perform a randomized controlled trial to evaluate the longitudinal feasibility and impact on outcomes that matter to caregivers. Specifically, we will focus on the caregiver’s sense of self-efficacy, mastery of caregiving, and stress and evaluate the frequency of app use over time and the types of features most used by caregivers.

## Appendix

Multimedia Appendix 1 Semistructured interview guide.

## Abbreviations

mHealth: mobile health
HIPAA: Health Insurance Portability and Accountability Act
TAM: Technology Acceptance Model

## Data Availability

The datasets generated during and/or analyzed during this study are available from the corresponding author on reasonable request.
