# Supplementary material for: An mHealth App to Support Caregivers in the Medical Management of Their Child With Cancer: Beta Stage Usability Study
Source: JMIR Cancer. 2024 Oct 17;10:e52128. doi: 10.2196/52128 (PMC11528164; doi:10.2196/52128)
Supplement: Multimedia Appendix 1 [file cancer_v10i1e52128_app1.docx]

*Thank you for agreeing to participate in this research project. The final goal of this project is to co-design and create an app to support caregivers of children with cancer in the medical management issues that occur in the home setting. Specifically, we want to help with symptom management, medication reminders and management, and symptom related medical knowledge. For this phase of the project, we are performing usability testing so that we can continue to refine the app and make it the best possible experience for caregivers of children with cancer.*

*Now that you have had access to the app for the last week, we would like to ask questions about specific features and aspects of the app. We will also leave time at the end for any questions or comments you would like to share.*

Setup:

1. How often did you use the app during the last week?
2. How was your experience with logging in to the app?
3. How was your experience with creating an account:
   1. For yourself?
   2. For your caregiver team?
   3. For your child with cancer?

Overall:

1. What was your overall impression of the Cope 360 app?
   1. What part of the app was the most helpful to you?
   2. Did you run into any issues while using the app?
   3. Do you think the app assisted you in the care of your child with cancer?

Symptom Tracking:

1. Did you use symptom tracking?
   1. What symptoms did you track? (Head, Temperature, Mouth & Throat, Back, Arms, Poop, Legs, Nausea & Vomit, Breathing)
   2. Did you find symptom tracking easy to use?
   3. What features of symptom tracking did you like?
   4. What were your thoughts on the time frames for reminding you to recheck a symptom?
   5. Did you run into any issues while using symptom tracking?
   6. What are your thoughts on the “pulsing heart”?
      1. Do you have a preference for a different way to show that a symptom is being tracked?
2. How did you go about stopping to track a symptom?
   1. Do you have any suggestions for how to stop a tracking event?

Emergency Planning:

1. Did you practice or use your emergency plan?
   1. Was your emergency plan easy to set up?
   2. Was your emergency plan helpful to you?
   3. Were there any issues with your emergency plan?
2. Did you have any additional thoughts to be added to the information related to when to seek care?

Conclusion:

1. Do you currently use any apps to care for your child with cancer?
   1. If yes, what apps and what do they do?
2. If this app was publicly available, would you continue to use it?
   1. If no, why?
3. Do you have any other thoughts you’d like to share with us on your experience using the COPE 360 app?

Generic probes:

- *What do you think was being represented here/there?*
- *Describe what you would do next/did do next.*
- *What about this picture/sentence/etc makes you feel that way?*
